# Assistive/Socially Assistive Robotic Platform for Therapy and Recovery: Patient Perspectives

**DOI:** 10.1155/2013/948087

**Published:** 2013-12-22

**Authors:** Matthew White, Mary Vining Radomski, Marsha Finkelstein, Daniel Allan Samuel Nilsson, Lars Ingimar Eugen Oddsson

**Affiliations:** Courage Kenny Research Center, Courage Kenny Rehabilitation Institute, part of Allina Health, Abbott Northwestern Hospital, Mail route 12212, 800 East 28th Street, Suite SK278, Minneapolis, MN 55407, USA

## Abstract

Improving adherence to therapy is a critical component of advancing outcomes and reducing the cost of rehabilitation. A robotic platform was previously developed to explore how robotics could be applied to the social dimension of rehabilitation to improve adherence. This paper aims to report on feedback given by end users of the robotic platform as well as the practical applications that socially assistive robotics could have in the daily life activities of a patient. A group of 10 former and current patients interacted with the developed robotic platform during a simulated exercise session before taking an experience-based survey. A portion of these participants later provided verbal feedback as part of a focus group on the potential utility of such a platform. Identified applications included assistance with reaching exercise goals, managing to-do lists, and supporting participation in social and recreational activities. The study participants expressed that the personality characteristics of the robotic system should be adapted to individual preferences and that the assistance provided over time should align with the progress of their recovery. The results from this study are encouraging and will be useful for further development of socially assistive robotics.

## 1. Introduction

Assistive robotics are often used in contemporary rehabilitation practice to support recovery from injury or condition-related impairments by helping patients perform exercises for which occupational and physical therapists typically provide hands-on assistance [1–4]. Socially assistive robotics provide noncontact user support and coaching to help patients adhere to home exercise and activity recommendations; this type of assistive robotics is at an earlier stage of development than robotics that involve physical interfaces and interactions with end users [5, 6]. 

Rehabilitative robotics has the potential to enhance adherence to rehabilitation recommendations, which is known to be difficult for those with chronic health conditions [7]. Research suggests that poor adherence compromises health outcomes [8], while high levels of therapy practice optimize motor recovery [9], underscoring the importance of strategies and technologies that bring rehabilitation support into patients' homes. Patients appear best able to follow through with rehabilitation exercise and activity recommendations with adherence support specifically tailored to their unique personal, social, and environmental requirements, enablers, and barriers [10]. By supporting each patient's requirements for at-home practice and implementation of therapy recommendations, assistive/socially assistive robotics may represent a low-cost, highly-accessible technology solution that improves rehabilitation outcomes.

Cognizant of these needs, a team of occupational/physical therapists, scientists, and engineers developed a prototype robotic system that included features of both assistive and socially assistive robotics, known as SKOTEE, the Sister Kenny hOme ThErapy systEm. The design objectives were to keep the system inexpensive, while providing for assisted exercise and help with adherence in the home environment. SKOTEE was designed to be used in the home environment to help patients perform their home exercise programs and provide reminders for exercising, appointments, and taking medications. The system includes an exercise module, reminders for exercises, appointments, and medications, a portal for messaging between patient and therapist, and means to play audio books for patient entertainment [11]. Therapists' interpretation of patients' needs informed initial prototype development, after which former patients used the system and provided input to guide further system refinements and possible modalities and domains. This approach recognizes the interrelationship between the individual and assistive technology as described by The Human Environment/Technology Interface Model (HETI) [12].

In this paper, we report on feedback and requirements reported by patient end users of SKOTEE. We had two primary study objectives: (1) to obtain feedback on the functionality of the SKOTEE from a group of patients with a diagnosis of stroke or neurological disorders, after having used it in a clinical setting, as well as eliciting suggestions regarding improvements and enhancements via a focus group and (2) from this same focus group, to learn about the needs of patients with a diagnosis of stroke who confront limitations on a daily basis due to their medical condition. During a two-hour focus group session we inquired about things they would like to do that they are not doing now or want to do differently with respect to managing their medical condition and daily activities as well as participating in social and recreational activities. Following this conversation, the focus group was reintroduced to the SKOTEE and was asked to provide suggestions as to how a SKOTEE-like platform could meet the needs identified during the first part of the session. The accomplishment of these two objectives complemented each other in providing a broader understanding of both, the preferred roles and characteristics of assisted and socially assistive robotics, from the patient perspective.

## 2. Materials and Methods

### 2.1. Participants

Ten patients, eight with stroke and two with neurologic impairments, some of whom were already discharged, were recruited to use the SKOTEE and provide feedback. Seven current and past patients with stroke diagnoses were recruited to participate in the focus group. Due to the noninterventional nature of the study, it was exempt by the Allina Health Institutional Review Board.

### 2.2. Technology

The first prototype was developed as part of a design project with mechatronics engineering students at the Royal Institute of Technology in Stockholm, Sweden and mechanical engineering students at the University of Minnesota. Their efforts were coordinated by a graduate student of the Royal Institute of Technology on site with research advisors from the Sister Kenny Research Center in Minneapolis where this study was conducted. The original prototype was built on the iRobot Create platform. It was subsequently upgraded to the ERA-MOBI platform.

We envisioned SKOTEE being used as both a stand-alone device providing verbal instructions to assist patients at home and as an assistive system using multiple exercise device modules that can interact wirelessly. The first exercise module prototype was designed to allow the patient with arm weakness and/or poor motor control to practice reaching motions such as shoulder flexion/extension, elbow flexion/extension, and internal/external rotation. This is accomplished through a height adjustable pole that connects to the base allowing two degrees of rotational freedom (Figure 2). Using two potentiometers at the base of the pole (similar to a joy stick), this module converts the position of the hand placed on the top of the pole into a digital signal that is sent to SKOTEE using Bluetooth wireless communication protocol allowing the patient to perform computer-mouse-like movements during the exercise games that are seen on the SKOTEE's LCD screen. The participants were exposed to two simple sample games during the hands-on session, erasing the screen to reveal an underlying picture and quickly move to specific targets in a polar coordinate system.

The SKOTEE is capable of moving around a room, locating a person, and prompting to do a physical exercise. SKOTEE also has the capability of delivering messages to their therapist. Therapists have the ability to track progress with the patient's exercise and make changes to the exercise program to increase the therapeutic challenge. From an entertainment perspective, SKOTEE can play audio books.

### 2.3. Obtaining Patient Input and Feedback

Two methods were used to obtain patient input and feedback regarding the SKOTEE system. Each end user completed an experience survey immediately after participating in a hands-on session with the technology. Approximately three months later, they participated in a focus group discussion.

### 2.4. Hands-On Session

**Figure 1:**
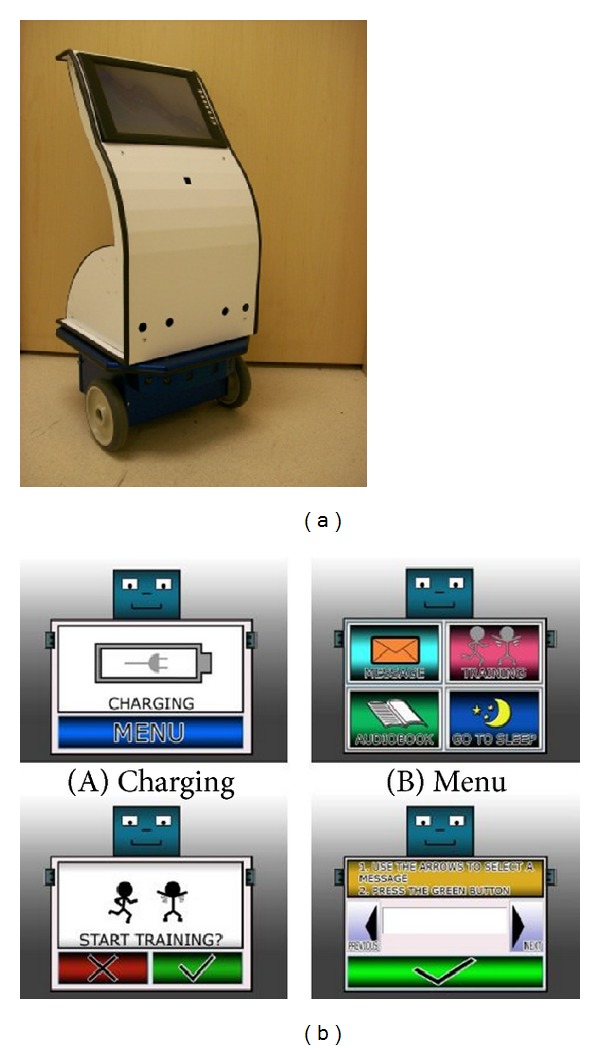
(a) SKOTEE prototype, a computer mounted on an upright mobile device that moves on two wheels. (b) Various screen shots from LCD screen.

The hands-on session was conducted in an area of the Courage Kenny Research Center containing furniture similar to a home family room environment, including a couch, chairs, a table, and work desk. Participants were able to interact with SKOTEE through an onboard touch screen. SKOTEE provided voice and screen commands and the participant responded by touching the screen (see Figure 1). An occupational therapist (Matthew White) was present during the hands-on session but did not intervene during the interaction with SKOTEE to guide the participants.

### 2.5. Experience Survey

The experience survey included questions relating to the patients' experiences using SKOTEE and other potential features. Each question was scored on a scale from 0 to 10, where 0 was the most negative score and 10 the most positive score. The survey also included a comment area for subjects to express their likes and dislikes and what they would want to see in future versions of SKOTEE.

### 2.6. Focus Group

**Figure 2:**
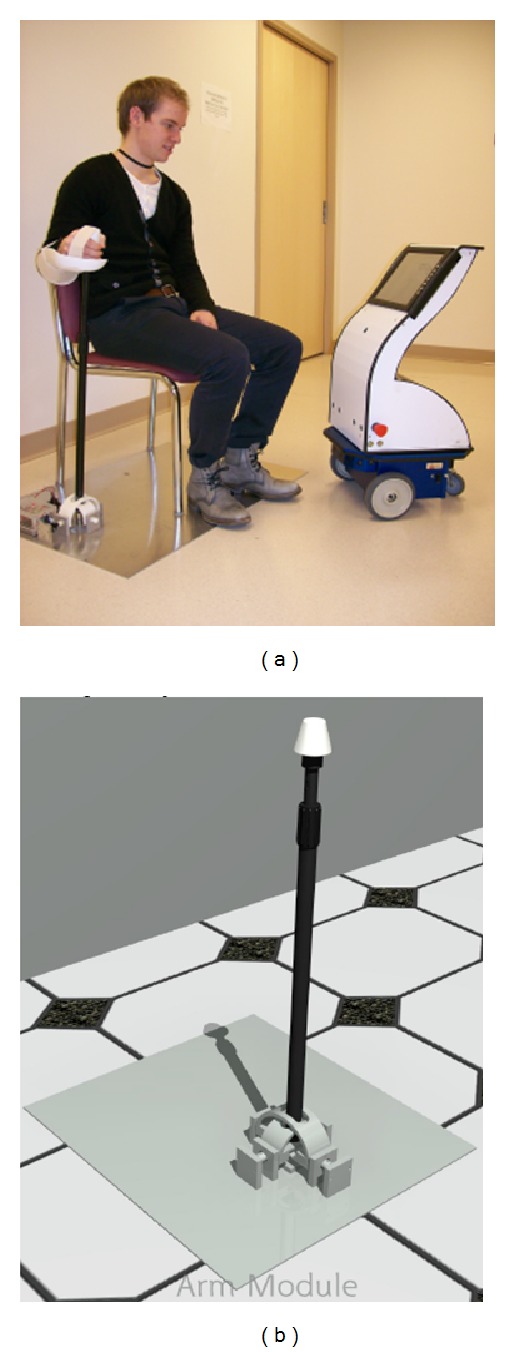
Arm module. (b) adjustable pole allowing 2 degrees of rotation type device. (a) Demonstrating user interacting with SKOTEE.

The focus group was conducted by an individual with experience in leading focus group discussions but who was not a member of the SKOTEE development team. An observer/transcriber took notes on paper posted to the walls of the room so that all participants could view them. The questions were designed by the group leader with input from the design team. The first part of the session was designed to identify the needs of the group with respect to managing their medical condition and every day activities with regard to remembering, tracking, and organizing as well as participating in social and recreational activities. The approach used was to first identify the types of technology and computer games the group was using. This inquiry would help set the stage for the second part of the session as well as identify and share, with other focus group members, use of these technologies in helping them meet their goals. Following this exploration, the group was asked to identify areas in which a personal coach could be helpful relative to medical conditions, remembering/tracking/organizing, and socialization/recreation. At the beginning of the second part of the session they received a demonstration of the SKOTEE via a video in addition to seeing the actual SKOTEE. It was suggested that they imagine SKOTEE as a platform that instead of the current embodiment (Figure 2) could run as an application on a computer, laptop or smartphone. They were then asked to circle back to the list of items generated for which a coach could be helpful and imagine how the SKOTEE could serve to meet these needs. Additionally, we sought their reaction to video conferencing/recording with a therapist receiving the output as well as their acceptance of the mobility of the robotic SKOTEE. Participants had access to the transcribed results during the session in order to stimulate additional discussion and help trigger additional responses based on coparticipant's viewpoints.

### 2.7. Analysis

Descriptive statistics were used to analyze the results of the experience survey. A simple descriptive analysis of the focus group results was based on transcribed responses to the questions.

## 3. Results

### 3.1. Objective 1: Functionality of the SKOTEE

Table 1 provides the median, minimum, and maximum scores for each of the questions included in the experience survey. Patients responded positively to most questions with median scores of at least 8.0 on a range of 0 to 10. The feedback provided for the helpfulness of the arm exercise received the lowest median score at 5.5 (range 3 to 9) and likeliness to rent received a median score of 6.5 (range: 2 to 10). Three of 10 subjects scored some survey items at values less than 5. Some comments included: “in early stages of rehab, I would have used it all the time, that's when I would see it being very beneficial,” “the accountability would be nice, reminder every day to do the exercise,” and “concerned about machine traveling around floor surfaces, around obstacles, thresholds.”

### 3.2. Objective 2: Focus Group

Table 2 describes the response to the first set of questions pertaining to participants' current use of technology in daily life. All participants used some form of computer or mobile device on a regular basis. Most participants played games on their computers or phones on a regular basis. They appeared to do so for both fun and for possible therapeutic benefit (exercise, balance).

In order to better understand the potential value of socially assistive robotics, participants were asked to explain if and how a personal coach might help them with everyday activities (Table 3). In the area of medical needs, participants emphasized their interest in better adhering to exercise goals. They appeared to understand what they needed to do but wanted their “personal coach” to hold them accountable to perform and provide feedback on performance. Participants had divergent preferences in the personality of their preferred personal coach ranging from drill sergeant to encourager to cheer leader. In the area of remembering/tracking, most participants used list making to keep track of information, with varying degrees of satisfaction. They thought that SKOTEE could integrate these lists, help with prioritization, and potentially decrease the need for handwriting. Most participants talked about SKOTEE acting as a reminder. In the area of socialization/recreation, participants appeared to toggle between wishes that their personal coach would locate suitable/desirable recreational options and provide physical assistance to actually support performance. Participants appeared to be interested in the possibility of using SKOTEE as a means of creating social interactions via an online community or even volunteering.

The final set of questions pertained to specific, potential SKOTEE features, video recording and platform. All of the participants saw the value of video recording interactive exercise sessions and having them shared with the therapist. A few expressed concern about privacy and wanted the assurance that the therapist would be the only individual who could view it. Two participants wanted to control what was sent to the therapist. Participants appeared to have divergent views as to SKOTEE's mobility feature (i.e., being able to travel around the room to the patient). Two individuals viewed the feature as acceptable (although, not enthusiastically) and four viewed the mobility feature as problematic and/or undesirable, preferring to have their “personal coach” housed in a multipurpose format (such as Ipad, Nintendo Wii, smartphone, etc.). Specifically, one participant stated she did not like it and would not want it. She felt that the SKOTEE would be in the way, they would possibly trip over it, and navigation would be difficult due to objects on the floor.

## 4. Discussion

We have shown that a robotic assist device is acceptable to patients with diagnoses of stroke or neurological problems. They felt safe using it, indicated that speech capability was important, rated the workout as good to very good, and thought that with the SKOTEE they would be motivated to do their arm exercise at home. When they were asked about rental of the SKOTEE, the median score at 6.5 was less than most other responses to other items on the experience survey at a median score of 8. The reluctance to rent may be related to the cost and/or the inconvenience of working with a rental company. This factor would need to be explored in designing a marketing and distribution plan. The lowest median score across all questions on the experience survey was 5.5 for the question about helpfulness of feedback from the arm exercise. This may be due to the inappropriateness of this specific exercise for some of the individuals who did not have a problem with their arm.

All participants in the focus group favorably endorsed the potential utility of a socially assistive robot that functioned as a personal coach. They identified three areas in which such a system would be helpful for (1) adherence to therapy recommendations, (2) organizing and remembering things to do, and (3) locating and supporting participation in social and recreational activities. While they agreed on the potential helpfulness of a socially assistive robot-coach, they varied widely in terms of the attributes or “personality” characteristics of said robot coach (some preferring a more aggressive sports persona, while others requiring a gentler, more encouraging persona). Beyond individually designed robotic persona requirements, participants indicated that functionality or focus of the “coach” should change over the course of their recovery. For example, some participants reported that it would have been helpful to receive hour-by-hour prompts from the “coach” to structure daily tasks immediately after hospital discharge whereas months later, they required reminders to perform intended daily exercise and to help them locate community activities that matched their interests and capabilities.

Participants' varying persona and functionality requirements aligns with assumptions about the necessity of an individualized approach to advance adherence to exercise and rehabilitation recommendations [10]. It also suggests that off-the-shelf consumer apps and computer games alone may not be sufficiently tailored to address individual needs and requirements. End user requirements support the potential benefits of socially assistive robotic platforms that can assume whatever persona will best engage the patient, perform changing and individually identified functions, and link to only those commercially available apps and games that match the patient's interests and capabilities.

There were several limitations to the study. Only 10 patients participated in the experience survey and seven in the focus group. It is possible that results would have been different with greater participation. Assessment of the arm exercise module was excellent for setup, motivating to exercise and level of workout, while usefulness of feedback was rated low at a median of 5.5. This may have been due to patients not having a problem with their arm and therefore the feedback was not perceived as useful. These results may have been different if patients were specifically selected who had a problem with their arm movement.

## 5. Conclusion

SKOTEE, a socially assistive robotics platform system may be a feasible tool to help patients adhere to rehabilitation in the home environment. Patients were overall positive to the concept and could see the benefit of a personal “coach” to help encourage and support their therapy needs. The system must be able to adapt and support different patient personalities. The form factor for the technology needs further exploration and development. An in-home feasibility study to explore changes in clinical outcomes appears justified.

## Figures and Tables

**Table 1:** Results of the experience survey (*N* = 10).

Experience survey question (scored 0 to 10 where higher is more positive)	Median	Minimum	Maximum
Technical: how safe you felt interacting with SKOTEE (not at all safe to very safe)	10	8	10
Technical: appearance (did not like at all to like a lot)	8.5	3	10
Technical: importance of speech capability (not at all important to very important)	8.5	3	10
Technical: use online environment to exercise (not likely to very likely)	9	3	10
Arm exercise: motivate to exercise (not at all to a lot)	9	2	10
Arm exercise: how easy to set yourself up (very difficult to very easy)	8	1	9
Arm exercise: level of workout (very poor to very good)	8	5	10
Arm Exercise: feedback (not at all helpful to very helpful)	5.5	3	9
Experience: use at home? (not at all likely to very likely)	9.5	4	10
Experience: beneficial to rehabilitation (not at all beneficial to very beneficial)	8.5	4	10
Experience: likely to rent? (not at all likely to very likely)	6.5	2	10
Experience: use on a daily basis (not at all likely to very likely)	9	2	10

**Table 2:** Use of technology in daily life.

Subject	Favorite technology	Technology used during a typical week	Computer games
1	MP3 (love music)	Laptop, smartphone, MP3	Card games and puzzles. No interactive games due to difficulty in visually tracking
2	Computer (access to any information I want)	Washing machine, laptop, nonsmart phone, and camera	Occasionally, X-Ball
3	Lower extremity stimulator (to walk)	Camera, lower extremity stimulator, IPOD, and tricycle, CD (read books)	Not any
4	IPOD, smart phone (computer in a pocket)	Computer, IPAD, IPOD, and smartphone	Wii-play with family. Used to improve balance and as a source for exercise.
5	Laptop computer, IPAD, IPhone	Laptop, IPAD, smartphone, and smart key for car	Suduko and BeJewel
6	Nook (to read)	Computer, X-Box	X-box (sports and adventure games)
7	Laptop computer and cell phone (for security)	Laptop, phone, walking aid, exercise equipment, and keyless entry to car	Not any except for solitaire occasionally on the phone

**Table 3:** Role of a personal coach and SKOTEE adaptation.

Subject	Needs	SKOTEE adaptation
Medical		
1	Keep me on task and focused and act as a cheerleader	(i) Remind the individual to perform tasks and exercises (ii) Providing a way of recording activity and exercise (iii) Remind the individual of their goals (iv) Provide reports of progress over time (v) Provide warnings if not using the activity recording mechanism as an indication of “no activity” (vi) Encourage good habits in doing exercises by reminding, especially at the beginning (vii) Provide positive feedback of meeting goals and doing exercises
2	Encourage to do exercise by reminding and answering Y/N questions	
3	Encourage to do exercise	
4	Set time aside to do exercise	
5	Keep accountable to perform tasks and exercise	
6	Encourage to do exercises	
7	Consistency in doing exercises, keep from procrastinating, and provide nutritional consulting	

Remembering and organizing		
1	Write “to dos” down
2	Breaking down a task
3	Remind about the steps of a task	(i) Provide voice recognition to create lists (ii) Remind of appointments through messaging, beeping, and calling (iii) Customize SKOTEE according to individual needs
4	Prioritize a list of tasks (to-dos) and identify what is a project versus a to-do task	
5	Break down projects (tasks) to simplify the approach, motivate progress, and acknowledge what has been completed	
6	Integrate lists that may be scattered and numerous
7	Remind of appointment and keep from losing track of time

Socialization/recreation		
1	Identify new things/activities to try out
2	Provide physical help to support participation
3	Provide help as needed to allow participation in outdoor activities	(i) Reminding (ii) Create a virtual support group (iii) Create a virtual volunteer network
4	Encourage to contact friends and arrange times to see them	
5	Help contact friends and family members	
6	Help participate in activities and encourage to “GO”
7	Allow individual to set pace and remind that it is okay and important to take time outs
